# *UBR4* deficiency causes male sterility and testis abnormal in *Drosophila*

**DOI:** 10.3389/fendo.2023.1165825

**Published:** 2023-07-10

**Authors:** Shi-Ming Xie, Jia-Xuan Lai, Chu-Qiao Liu, Xi-Xing Zhang, Yong-Miao Lin, Qi-Wen Lan, De-Yao Hong, Xiao-Chuan Chen, Jing-Da Qiao, Yu-Ling Mao

**Affiliations:** ^1^ Department of Neurology, Institute of Neuroscience, Key Laboratory of Neurogenetics and Channelopathies of Guangdong Province and the Ministry of Education of China, The Second Affiliated Hospital, Guangzhou Medical University, Guangzhou, China; ^2^ The First Clinical Medicine School of Guangzhou Medical University, Guangzhou, China; ^3^ The Second Clinical Medicine School of Guangzhou Medical University, Guangzhou, China; ^4^ Department of Obstetrics and Gynecology, The Sixth Affiliated Hospital, Sun Yat-sen University, Guangzhou, China; ^5^ Department of Obstetrics and Gynecology, Center for Reproductive Medicine, Guangdong Provincial Key Laboratory of Major Obstetric Diseases, The Third Affiliated Hospital of Guangzhou Medical University, Guangzhou, China; ^6^ Key Laboratory for Reproductive Medicine of Guangdong Province, The Third Affiliated Hospital of Guangzhou Medical University, Guangzhou, China

**Keywords:** male fertility, hatchability, drosophila, testes, UBR4

## Abstract

**Introduction:**

It has been established that *UBR4* encodes E3 ubiquitin ligase, which determines the specificity of substrate binding during protein ubiquitination and has been associated with various functions of the nervous system but not the reproductive system. Herein, we explored the role of *UBR4* on fertility with a *Drosophila model*.

**Methods:**

Different *Ubr4* knockdown flies were established using the UAS/GAL4 activating sequence system. Fertility, hatchability, and testis morphology were studied, and bioinformatics analyses were conducted. Our results indicated that *UBR4* deficiency could induce male sterility and influent egg hatchability in *Drosophila*.

**Results:**

We found that *Ubr4* deficiency affected the testis during morphological analysis. Proteomics analysis indicated 188 upregulated proteins and 175 downregulated proteins in the testis of *Ubr4* knockdown flies. Gene Ontology analysis revealed significant upregulation of *CG11598* and *Sfp65A*, and downregulation of *Pelota* in *Ubr4* knockdown flies. These proteins were involved in the biometabolic or reproductive process in *Drosophila*. These regulated proteins are important in testis generation and sperm storage promotion. Bioinformatics analysis verified that *UBR4* was low expressed in cryptorchidism patients, which further supported the important role of *UBR4* in male fertility.

**Discussion:**

Overall, our findings suggest that *UBR4* deficiency could promote male infertility and may be involved in the protein modification of *UBR4* by upregulating *Sfp65A* and *CG11598*, whereas downregulating Pelota protein expression.

## 1 Introduction

Current evidence suggests that maintaining cellular protein homeostasis (proteostasis) largely relies on proteolysis (1). As a cellular mechanism that triggers proteasomal degradation, ubiquitin conjugation, based on ubiquitin and ubiquitin-like proteins (UBLs), plays an important role in controlling cell division, signal transduction, embryonic development, endocytic trafficking, and immune response (2).

*UBR4* encodes the Ubiquitin Protein Ligase E3 Component N-Recognin 4; the homology of *Drosophila* is *poe*. It is an E3 ubiquitin ligase (E3 enzyme) that regulates protein degradation, removing damaged or misfolded proteins from the cellular environment, thereby regulating cellular homeostasis. The body utilizes the coordinated action of a series of enzymes in the ubiquitin–proteasome system (UPS), including ubiquitin-activating enzymes (E1 enzymes), ubiquitin-conjugating enzymes (E2 enzymes), and E3 ubiquitin ligases (E3 enzymes) (1–4). E3 enzymes are reportedly important in the ubiquitination process and determine substrate specificity (3). Protein error correction plays an extremely important role in physiological processes such as growth and development, reproductive inheritance, immunity, and endocrine of *Drosophila* and the human body.

Up to now, research on *UBR4* has mainly focused on the nervous system (5–7). Interestingly, the number of *Drosophila* pupae decreased after *Ubr4* gene knockdown in our preliminary experiments, suggesting that *Ubr4* may be related to reproductive capacity. This phenomenon may be attributed to erroneous protein synthesis in the genitals, which may cause physiological and behavioral changes, including reduced egg production and feeding, decreased willingness to mate and storage/utilization of sperm, and altered gene expression (8, 9). Spermatogenesis abnormality is also another important reason for reproductive problems. In *Drosophila*, spermatogenesis starts at the apical tip of the testis, which hosts two stem cell populations, including the germline stem cells (GSCs) and somatic cyst stem cells (CySCs) (10–12). Zygotes produced by GSCs undergo four rounds of mitosis and then differentiate into 16 spermatogonia. The spermatogonia then differentiates to spermatocytes, round spermatozoa, long spermatozoa, and mature spermatozoa and complete an entire spermatogenesis process. The cyst cells differentiated from CySCs encase germ cells and support germ cell growth (13). The hub cells mainly maintain the self-renewal and differentiation of GSCs and CySCs (14, 15).

Herein, loss-of-function *Drosophila* models were applied as research vectors to detect the effect of the *Ubr4* gene on *Drosophila* development and reproduction.

## 2 Materials and methods

### 2.1 Fly culture and qPCR

*Drosophila* was maintained on standard cornmeal and incubated under controlled conditions (temperature 25°C, humidity 60%–70%, and 12:12-h light/dark cycle). *UAS-Ubr4-RNAi* (THU1137, CG14472) was donated by Tsing Hua Fly Center (Tsinghua University, Beijing, China). The knockdown efficiency of RNAi was checked by qPCR (16). The primer sequencies were

Ubr4/Poe-F1: CCACCGTCACACACTTCAAT
Ubr4/Poe-R1: GGCAGCAGTCCATTACATCT
GAPDH-F1: CGTCAACGATCCCTTCATCGATGTC
GAPDH-R1: CAGCACTGGCCCAGTTGATGTTG.

### 2.2 Fertility and hatchability assays

The quantification of fertility and hatchability: Fertility refers to the ability of *Drosophila* to produce offspring, and our study mainly focused on the number of eggs it laid. Hatchability is the rate of eggs hatching into pupae and pupae hatching into adults. The details of analyses were shown below.

#### 2.2.1 *Ubr4* knockdown male fertility and hatchability assay

*UAS-Ubr4-RNAi* males were mated with *tub-GAL4* females to generate *Ubr4* knockdown flies (*tub-GAL4>UAS-Ubr4-RNAi*). *UAS-Ubr4-RNAi* males were used as their genetically matched controls. Subsequently, 3-day-old virgin knockdown and control males, isolated within 24 h of their eclosion, were mated with 3–5-day-old wild-type (*Canton-S*) virgin female flies for different assays, as described below.

Three males were mated with *Canton-S* virgin females in pairs. Then, males were discarded after 48 h. Mated females were allowed to lay eggs for 10 days with the change to fresh food vials at 48-h intervals for the fertility assay. The number of eggs laid, egg-to-pupa hatchability rate, and pupa-to-adult hatchability rate was calculated. Mated females were discarded after 10 days, and the resultant progenies were counted to determine fertility. For each group, the experiments were repeated three times with five to six replicates each.

#### 2.2.2 *Ubr4* knockdown female fertility and hatchability assay

Three-day-old *Ubr4* knockdown (*tub-GAL4>UAS-Ubr4-RNAi*) and control (*UAS-Ubr4-RNAi*) virgin females, isolated within 24 h of their eclosion, were mated with 3–5-day-old Canton-S males for fertility assays.

After three males were mated with *Canton-S* virgin females in pairs, the males were discarded. We allowed mated females to lay eggs for 10 days, with fresh food vials changed every 48 h for the fertility assay. A fertility test was carried out by counting the progeny of females discarded after 10 days of mating. Hatchability is defined the same as above.

### 2.3 Larva development experiment

Newly *Ubr4* knockdown (*tub-GAL4>UAS-Ubr4-RNAi*) and control (*UAS-Ubr4-RNAi>Canton-S*) instar larvae cultured in standard medium for 24, 48, 72, 96, and 120 h after egg laying (AEL) were heated with 40°C–50°C water for 15 min to foster the larvae to come out from the medium. Afterward, the collected larvae were washed with phosphate-buffered saline (PBS), and their length was measured under the microscope. Next, the number of larvae developing into pupae was counted at 6-h intervals each day until all larvae had hatched. Subsequently, the number of adults hatched from pupae at 6-h intervals was counted. At the same time, the time of hatching from larvae to pupae and pupae to adults were counted independently.

### 2.4 Testes morphology

*Drosophila* testes from 2-day-old young adults were dissected in 1× PBS, fixed in 1× PBS/4% PFA for 30 min at room temperature. After being washed three times with PBS, the testes were transferred to glass slides. A mounting medium with 4′,6-diamidino-2-phenylindole (DAPI) (Abcam, USA) was dripped and reacted with the testes for 10 min before being covered with a coverslip. Testes morphology was observed with an inverted phase-contrast microscope (SP8; Zeiss, Jena, Germany). The number of spermatogenic cells was counted by ImageJ software (National Institutes of Health, Bethesda, MD, USA), and data were analyzed by GraphPad Prism 7.0.

### 2.5 Mass spectrometry and bioinformatics analyses

#### 2.5.1 Extraction of protein and enzymatic hydrolysis of peptides

Testes were collected and frozen in liquid nitrogen before protein extraction. Tissue protein was extracted by SDT (4% (w/v) SDS, 100 mm Tris/HCl pH 7.6, 0.1 m DTT), then protein was quantified by BCA. AFign appropriate amount of protein was obtained from each sample, and enzymatic hydrolysis of trypsin was performed by filter-aided proteome preparation (FASP). Next, the peptide was desalted by the C18 Cartridge. After freeze-drying, the peptide was lyophilized and redissolved in 40 μl of 0.1% formic acid solution (OD280).

#### 2.5.2 Protein identification and quantitative analysis

The MaxQuant software (version number 1.6.14) (17) was used for database identification and quantitative analysis. Mass spectrometry was carried out by Shanghai Applied Protein Technology Co., Ltd.

#### 2.5.3 Bioinformatics analyses

The CTD (Comparative Toxicogenomics Database), which can be used for the prediction of correlations between genes, diseases, and chemicals (18), indicates that changes in *UBR4* may be associated with cryptorchidism (http://ctdbase.org/). As one of the reasons for male infertility disorders, cryptorchidism, teratospermia, and azoospermia may be risk factors for the disease (19). To validate the findings, we downloaded the cryptorchidism dataset (GSE16191), teratospermia (GSE6872), and azoospermatism (GSE108886) from the GEO (Gene Expression Omnibus) database. With the screening criteria that (1) there were clear experimental and control groups, (2) the experimental group did not have any treatment other than cryptorchidism, (3) the samples were all from humans, and (4) the dataset had been borrowed from other articles, we finally chose the cryptorchidism dataset GSE16191 (20) and completed a bioinformatics analysis using R software (R.4.2.2), containing 16 diseased samples and 4 healthy control samples with tissue taken from whole testes (http://www.ncbi.nlm.nih.gov/geo/). The expression of three key genes, *UBR4*, *PELO*, and *LIPA*, was compared in groups to validate their up- and downregulation, and ROC curve analysis of key genes was performed to predict their diagnostic efficacy for cryptorchidism. A single-gene GSEA was performed to identify downstream genes in the functional pathway of the key genes. GO and KEGG analyses of downstream genes and key genes were also performed to search for related biological processes and enrichment pathways mediated by key genes.The quantitative information of the target protein set was normalized (normalized to (-1,1) interval). Then, the ComplexHeatmap R package (R Version 3.4) was used to classify the two dimensions (distance algorithm: Euclid, connection mode: average linkage) of the expression of samples and protein simultaneously. Finally, a hierarchical clustering heat map was generated.

GO annotation of the target protein set by Blast2GO can be summarized into four steps: sequence comparison (Blast), GO item mapping, GO annotation, and InterProScan annotation. The ProScan software package, which runs a scanning algorithm from the InterPro database in an integrated way to characterize the sequence, was used to obtain the domain annotation information of the target protein sequence in the Pfam database.

### 2.6 Statistical analysis

All quantitative data were expressed as mean ± SD. The Student’s t-test was used to compare two independent or paired samples. Multiple samples were compared using one-way ANOVA, and differences between the two groups were evaluated using Tukey’s *post-hoc* test. The data were analyzed using GraphPad Prism 7.00 and SPSS 20. A P-value < 0.05 was statistically significant.

## 3 Results

### 3.1 *Ubr4* deficiency influences larval development

**Figure 1:**
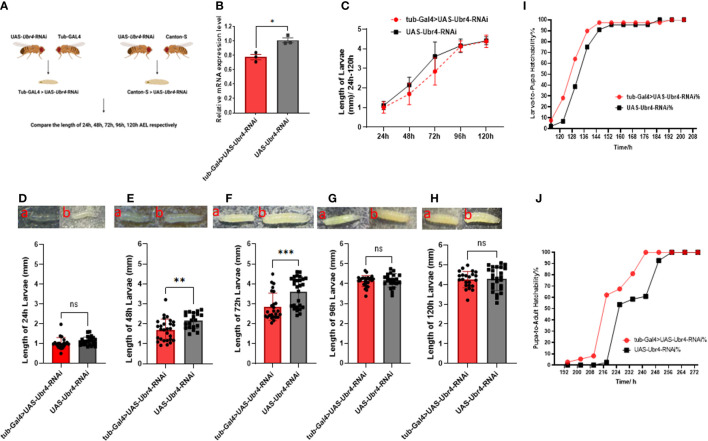
Comparison of larval development between *Ubr4* knockdown and wild type. **(A)** Experimental procedure. **(B)** Relative mRNA expression level in WT and *Ubr4* knockdown flies. **(C)** The overall length variation (24–120 h) of larvae. **(D)** Comparison of the length of 24-h-old larvae. **(E)** Comparison of the length of 48-h-old larvae. **(F)** Comparison of the length of 72-h-old larvae. **(G)** Comparison of the length of 96-h-old larvae. **(H)** Comparison of the length of 120-h-old larvae. a. *tub-Gal4>UAS-Ubr4-RNAi*, b. *UAS-Ubr4-RNAi*. **(I)** Larval hatchability at different time points. **(J)** Pupae hatchability at different time points. ns, P>0.05, *P<0.05, **P<0.01, ***P<0.005.

Flies crossing strategy in larval development assay was showed in **Figure 1A**. The Knockdown efficiency of RNAi is about 20% (**Figure 1B**). Compared with the control group, the length of *tub-GAL4>UAS-Ubr4-RNAi* larvae was lower than that of *UAS-Ubr4-RNAi> Canton-S*, especially for 48-h AEL and 72-h AEL. The length of 48h AEL *tub-GAL4>UAS-Ubr4-RNAi* larvae was significantly lower than that of *UAS-Ubr4-RNAi> Canton-S* (1.694 ± 0.544 mm [n = 28] vs. 2.159 ± 0.388 mm [n = 20]; **P = 0.002, **Figure 1E**). At the same time, the 72-h AEL *tub-GAL4>UAS-Ubr4-RNAi* larvae had a lower length than *UAS-Ubr4-RNAi>Canton-S* (2.847 ± 0.687 mm [n = 25] vs. 3.608 ± 0.744 mm [n = 27]; ***P = 0.0004, **Figure 1F**). However, when the larvae developed to 24-h AEL, 96-h AEL and 120-h AEL, there was no significant difference in larval length between the *tub-GAL4>UAS-Ubr4-RNAi* and *UAS-Ubr4-RNAi> Canton-S* flies (24-h AEL: 1.007 ± 0.292 m24-h AEL: 1.007 ± 0.292 mm [n = 21] vs. 1.112 ± 0.211 mm [n = 25]; P >0.05 Figure24-h AEL: 1.007 ± 0.292 mm [n=21] vs. 1.112 ± 0.211 mm [n=25]; P>0.05, **Figure 1D**. 96-h AEL: 4.125 ± 0.276 mm [n = 26] vs. 4.157 ± 0.340 mm [n = 21]; P > 0.05, **Figure 1G**. 120-h AEL: 4.253 ± 0.417 mm [n = 23] vs. 4.258 ± 0.588 mm [n = 25]; P > 0.05, **Figure 1H**). These results suggested that the heterogeneity in developmental speed accounted for the difference in larval length observed in our study.

Further experiments showed that there was no significant difference in the development time from larva stage to pupa and from pupa stage to adult between the *tub-GAL4>UAS-Ubr4-RNAi* and *UAS-Ubr4-RNAi> Canton-S* groups (**Figures 1I, J**).

### 3.2 *UBR4* is essential for male fertility but not female fertility

**Figure 2:**
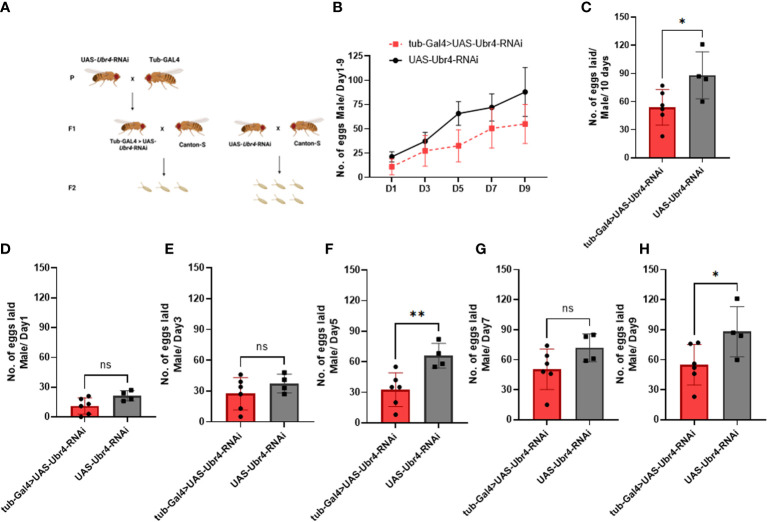
Reproductive performance of females mated to *Ubr4* knockdown males. **(A)** Experimental procedure. **(B)** The overview of eggs laid from days 1 to 9. **(C)** The overall fertility (total no. of eggs laid/10 days) of mated females. **(D)** Day 1 fertility (no. of eggs laid/day from day 1) of mated females. **(E)** Day 3 fertility (no. of eggs laid/day from day 3) of mated females. **(F)** Day 5 fertility (no. of eggs laid/day from day 5) of mated females. **(G)** Day 7 fertility (no. of eggs laid/day from day 7) of mated females. **(H)** Day 9 fertility (no. of eggs laid/day from day 9) of mated females. ns, P>0.05, *P<0.05, **P<0.01.

Wild-type females (*Canton-S*) mated to *Ubr4* knockdown males (*tub-Gal4>UAS-Ubr4-RNAi*) produced significantly fewer fertilized eggs than those mated with wild-type (*UAS-Ubr4-RNAi*) males. The reduction in fertility observed in *Drosophila* could be due to several reasons, including fewer eggs being laid by mated females and/or reduced hatchability/increased mortality of these laid eggs during development (21). Therefore, to determine the cause of decreased fertility of *Ubr4* knockdown males, we counted the number of eggs laid (fertility) on day 1, day 3, day 5, day 7, and day 9. Flies crossing strategy was shown in **Figure 2A**. **Figure 2B** showed the overview of eggs numbers of Ubr4 knockdown group and control group. Over 10 days, the number of eggs produced by the females mated with *tub-GAL4>UAS-Ubr4-RNAi* males was less than in those mated with *Canton-S>UAS-Ubr4-RNAi* males (53.83 ± 18.90 [n = 6] vs. 88.00 ± 25.10 [n = 4]; *P = 0.0388, **Figure 2C**), on day 5 (32.50 ± 16.53 [n = 6] vs. 65.75 ± 12.18 [n = 4]; **P = 0.0090, **Figure 2F**) and day 9 (55.00 ± 20.26 [n = 6] vs. 88.00 ± 13.90 [n = 4]; *P = 0.05, **Figure 2H**). No significant difference was shown on day 1, day 3, day 7 between the tub-Gal4>UAS-Ubr4-RNAi and UAS-Ubr4-RNAi> Canton-S flies (P>0.05, **Figures 2B**, **2D**, **2G**).

**Figure 3:**
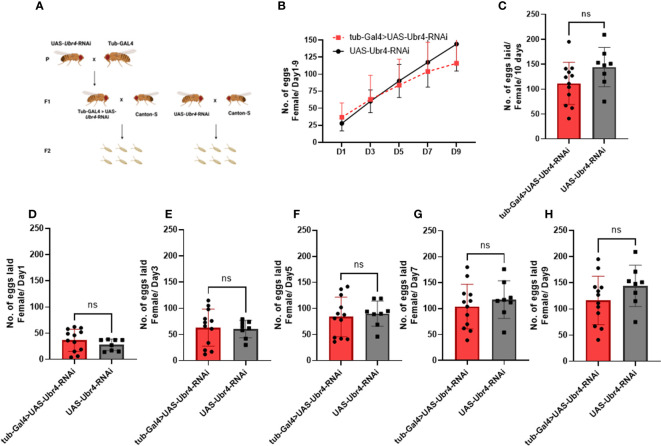
Reproductive performance of *Ubr4* knockdown females mated to wild males. **(A)** Experimental procedure. **(B)** The overview of eggs laid from days 1 to 9. **(C)** The overall fertility (total no. of eggs laid/10 days) of mated females. **(D)** Day 1 fertility (no. of eggs laid/day from day 1) of mated females. **(E)** Day 3 fertility (no. of eggs laid/day from day 3) of mated females. **(F)** Day 5 fertility (no. of eggs laid/day from day 5) of mated females. **(G)** Day 7 fertility (no. of eggs laid/day from day 7) of mated females. **(H)** Day 9 fertility (no. of eggs laid/day from day 9) of mated females. ns, P>0.05.

However, after wild-type males (*Canton-S*) were mated to *Ubr4*-deficient females (*tub-Gal4>UAS-Ubr4-RNAi*), no significant difference was observed compared with the control (*Canton-S* males mated with *UAS-Ubr4-RNAi*) in the overall number of progenies produced over 10 days (P > 0.05, **Figures 3B–H**). Therefore, the *Ubr4* gene has a more important influence on male fertility than on female fertility.

**Figure 4:**
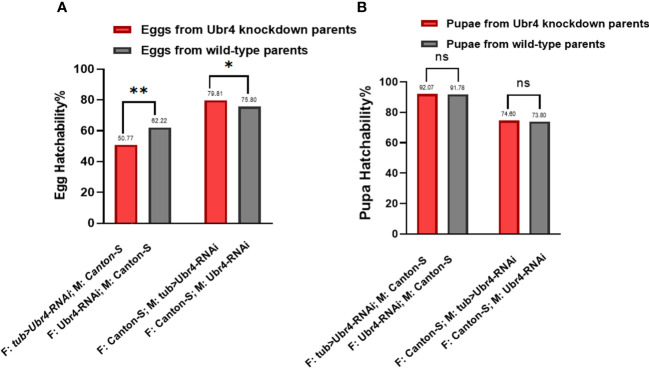
Egg hatchability and pupa hatchability. The hatchability rate was analyzed in two aspects, egg hatching rate **(A)** and pupa-to-adult rate **(B)**. Two gender groups were included: one was *Ubr4* knockdown males mated to wild females, and the other was *Ubr4* knockdown females mated to wild males. F, father; M, mother. ns, P>0.05, *P<0.05, **P<0.01.

To determine hatchability, we focused on the number of eggs that grew into pupae (egg hatchability) and the pupae that developed into adults (pupae hatchability). Analysis of the hatchability showed no significant difference in pupa-hatching rates between the pupae from Ubr4 knockdown parents and the pupae from wild-type parents (**Figure 4B**). In the egg hatchability, the hatching rate of the eggs reproduced from wild-type mothers with *Ubr4* knockdown fathers is lower than that from wild-type parents (**P = 0.0090, **Figure 4A**), whereas the hatching rate of eggs reproduced form *Ubr4* knockdown mothers with wild-type fathers is higher than that from wild-type parents (*P = 0.0175, **Figure 4A**).

### 3.3 *Ubr4* deficiency affected testis morphology

Morphological changes in *Ubr4* knockdown flies were studied by confocal imaging. DAPI, a well-established blue-emitting fluorescent compound, was used to visualize the cell nucleus. The testis cells of the Ubr4 knockdown flies appear slightly obscure compared with wild-type flies, which required stronger laser power to be visualized (550 vs. 480). In general, the Hub cells, CySCs, GSCs, and Sp labeled by DAPI are easier to be observed whereas the spermatocytes and cyst cells are not easy to be observed. Thus, knockdown of Ubr4 may alter the ratio of cell types in the testis. However, further experiments with a cell-type-specific antibody should be used for studying which stage of germ cells or what kind of somatic cells are affected.

**Figure 5:**
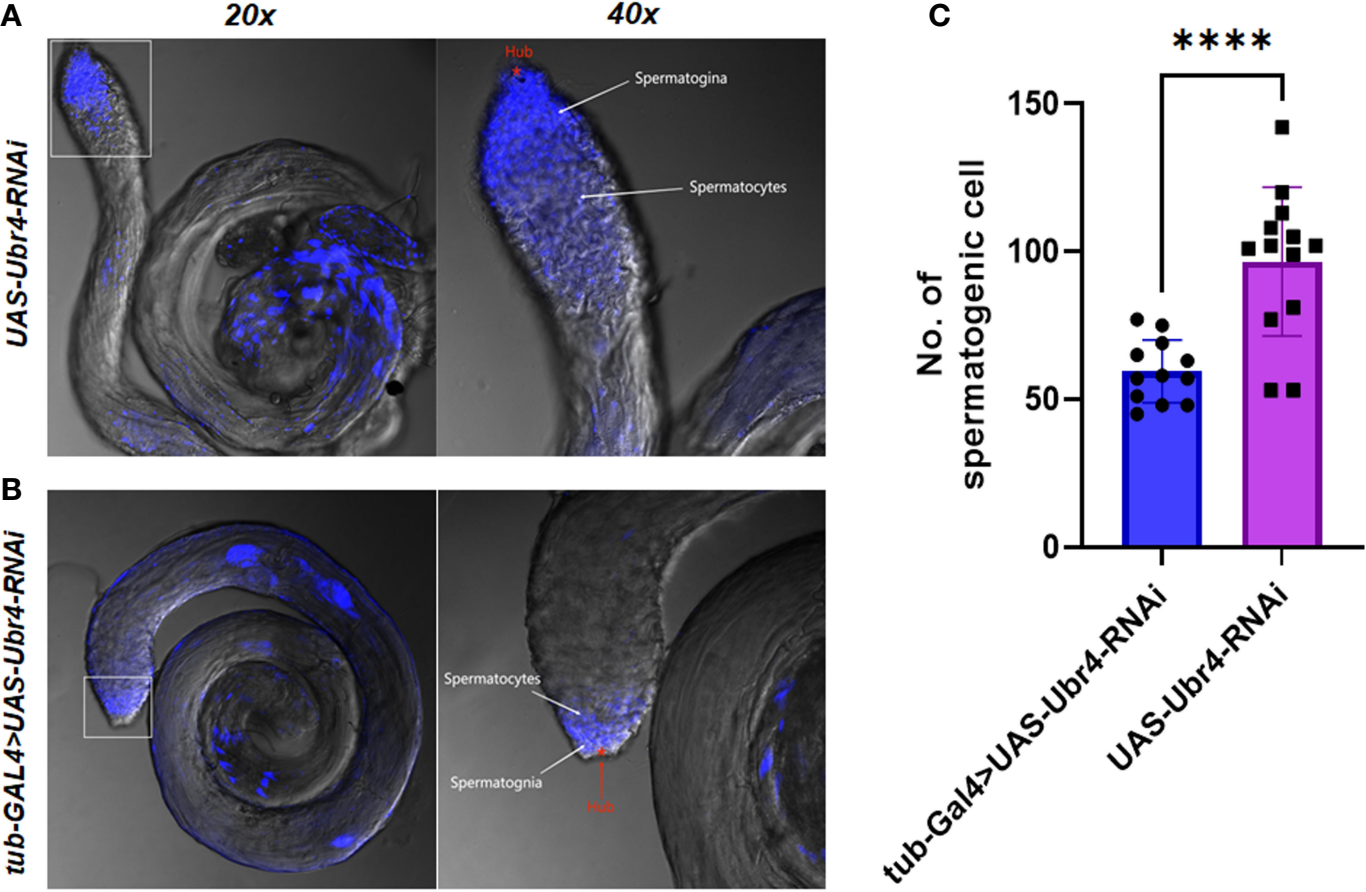
Testis morphology and spermatogenic cells To assess the effect of *Ubr4* deficiency, testes were analyzed at the ultrastructural level. Images showing the structure of WT *Drosophila* male testis **(A)** and *Ubr4* knockdown *Drosophila* **(B)**. **(C)** Quantity of spermatogenic cells in testis. ****P<0.001.

Furthermore, we tried to identify the number of spermatogenic cells in the germinal center of each group. The whole testes can be observed under a 20× confocal microscope, and spermatogonia at various stages were visible with 40× magnification (**Figures 5A, B**). The testes in the *Ubr4* knockdown group had a collapsed morphology and a disorganized internal structure compared with the control group. The number of spermatogenic cells was analyzed by ImageJ. Statistical results showed that there was a significant difference in the number of spermatogenic cells near the head of the testis (59.42 ± 10.62 [n = 12] vs. 96.62 ± 25.15 [n = 13]; ****P < 0.0001, **Figure 5C**).

### 3.4 *Pelota* was downregulated, whereas *CG11598* and *Sfp65A* were upregulated in the testis of *Ubr4*-deficient flies

**Figure 6:**
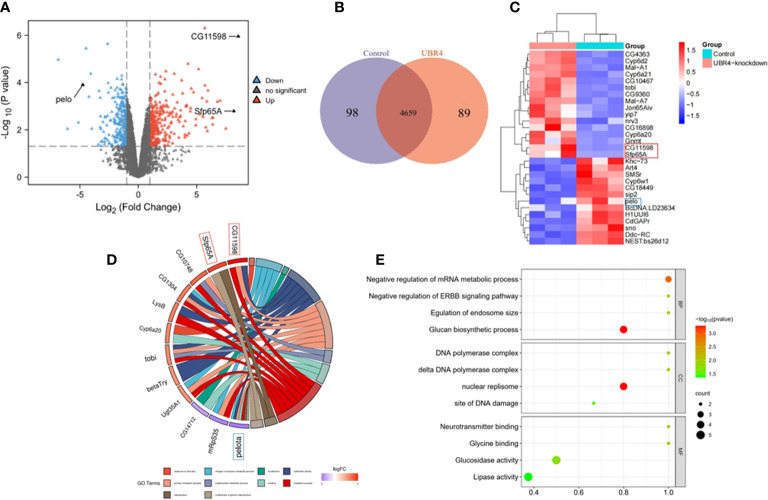
Mass spectrometry and bioinformatics analyses **(A)** Volcano plot showing the differentially expressed proteins from the WT control and the *Ubr4* knockdown group in the testis of *Drosophila*. The vertical lines mark the rate of the *Ubr4*-knockdown group compared to the control group, and the horizontal lines mark the P value. The upper right quadrants show the proteins upregulated compared to the control group, and the upper left quadrants contain downregulated compared to the control group. **(B)** Proteomics data show significant differences between WT and *Ubr4*-knockdown *Drosophila* testis in terms of upregulated and downregulated proteins. **(C)** The heatmap displays the difference in protein expression between *Ubr4*-knockdown and WT controls (P < 0.05). **(D)** GO enrichment analysis of the above differentially expressed proteins highlighted that the upregulation of *Sfp65A* and downregulation of *Pelota* were involved in the reproductive process. **(E)** GO enrichment analysis of key targets. It included Biologic Process (BP), Cellular Component (CC), and Molecular Function (MF).

To better understand the differential protein expression patterns between WT and *Ubr4*-knockdown testis, the mechanisms underlying *Ubr4*-knockdown-induced lower reproduction capacities in male *Drosophila* were explored by label-free proteomics. There were 363 differentially expressed proteins between WT and *Ubr4* knockdown *Drosophila* (**Figure 6A**). In comparison with WT control *Drosophila*, we found 188 upregulated proteins and 175 downregulated proteins in *Ubr4*-knockdown *Drosophila* (P < 0.05, **Figure 6B**). Heatmaps showed the alterations in the expression of 363 proteins (**Figure 6C**). Protein nodes that were upregulated (above 2.0-fold expression, red color) or downregulated (below 0.5-fold expression, blue color) were displayed using a gradient coloring scheme. Among these 363 proteins, CG11598 (orthologous to human LIPA) was the most upregulated and Sfp65A was the second, whereas Pelota (orthologous to human PELO) exhibited a significant downregulation after *Ubr4* knockdown (**Figure 6C**). Next, we conducted GO annotation of the 363 differential proteins affected by the knockdown of *Ubr4* to determine their function. It was suggested that the upregulation of *Sfp65A* and downregulation of *Pelota* were associated with GO terms Reproduction and Multicellular organism reproduction (**Figure 6D**), related to the reproductive process in *Drosophila*. *CG11598* was related to GO terms Metabolic process, Primary metabolic process, and Hydrolase activity, which suggested that upregulation of CG11598 may reduce male fertility through biometabolic processes. The number of differential proteins was counted at the GO secondary functional annotation level, including Biologic Process (BP), Cellular Component (CC), and Molecular Function (MF). The following are related to the GOs most affected by the loss of *Ubr4*. The delta DNA polymerase complex and DNA polymerase complex were mainly enriched in CC. Regulation of endosome size, negative regulation of ERBB signaling pathway, and negative regulation of the mRNA metabolic process were significantly enriched in BP by the mutant of *Ubr4*. Glycine binding, neurotransmitter binding, glucosidase activity, and lipase activity enriched in MF also showed an important interaction after knockdown of *Ubr4* (**Figure 6E**).

### 3.5 The function of *UBR4* tested and verified with bioinformatics analyses

*CG11598* is orthologous to human *LIPA*, and *Pelota* is orthologous to human *PELO*. *Sfp65A* has no homologous gene in human. Therefore, this study focuses on the expression of *UBR4*, *LIPA*, and *PELO* in the cryptorchidism dataset.

**Figure 7:**
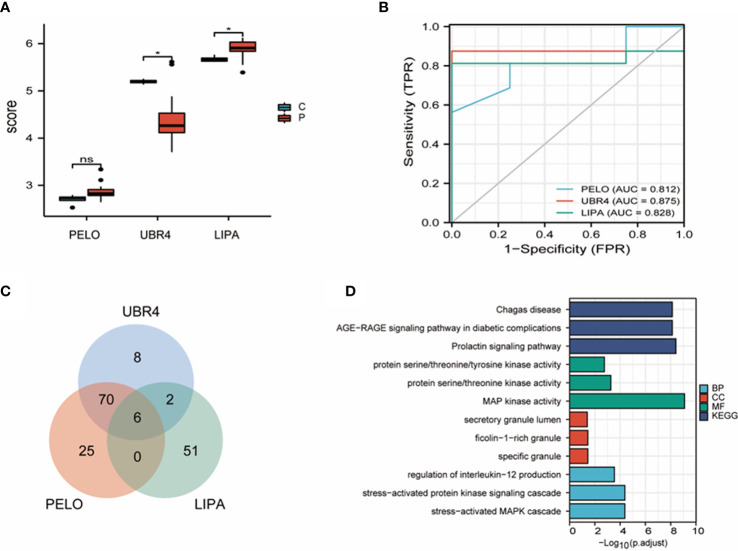
Bioinformatics analyses of *UBR4* and its related genes. **(A)** Box plot for expression control of *UBR4*, *LIPA*, and *PELO*. **(B)** Two hub genes were applied for the ROC (Receiver Operating Characteristic) evaluation in GSE16191. **(C)** Six downstream genes of the hub genes (*UBR4*, *LIPA*, and *PELO*) were selected using a Venn plot to visualize the single-gene GSEA (gene set enrichment analysis). **(D)** The GO\KEGG analysis of the hub genes and the downstream genes. ROC, receiver operating characteristic; GSEA, gene set enrichment analysis; GO, Genetic Ontology; KEGG, Kyoto Encyclopedia of Genes and Genomes. ns, P>0.05, *P<0.05.

Expression grouping box plots showed good between-group differences for *UBR4* (*P = 0.02) and *LIPA* (*P = 0.049) but confirmed that the between-group differences for *PELO* were not statistically significant (P = 0.06), and the up- and downregulation were consistent with the experimental results (**Figure 7A**). ROC curve analysis (**Figure 7B**) showed relatively high diagnostic efficacy for all three genes (AUC > 0.8). Single-gene GSEA predicted key genes downstream, and six results were screened (*MAPK14*, *NFKB1*, *MAPK13*, *MAPK11*, *MAPK9*, and *PIK3CD*) (**Figure 7C**). GO analysis showed that BP was mainly enriched in the regulation of interleukin-12 production, stress-activated protein kinase signaling cascade, and stress-activated MAPK cascade. MF is mainly enriched in protein serine/threonine/tyrosine kinase activity and MAP kinase activity. CC is predominantly enriched in the secretory granule lumen and is enriched in ficolin-1-rich granule and specific granule. KEGG was mainly enriched in Chagas disease, AGE-RAGE signaling pathway in diabetic complications, and prolactin signaling pathway (**Figure 7D**).

## 4 Discussion

Infertility is characterized by failure to establish a clinical pregnancy after 12 months of regular and unprotected sexual intercourse and is estimated to affect between 8% and 12% of reproductive-aged couples worldwide. Males are responsible for 20%–30% of infertility cases but contribute to 50% of cases overall (22, 23). Therefore, it is important to understand the mechanisms underlying fertility to improve our management of this patient population. In the present study, animal experiments indicated that *UBR4* represented a potential infertility gene that could induce infertility and abnormal testis structure in male *Drosophila*.

It is well-established that *UBR4* plays a role in the nervous system (24). To our knowledge, little study has hitherto assessed the role of *Ubr4* in the reproductive capability of *Drosophila* (25). Herein, we used bioinformatics and fly models to further confirm its important role in fertility. We provided compelling evidence that *Ubr4* deficiency could induce a decline in male reproductive capacity and abnormal testis structure. Intriguingly, no significant difference was observed in egg production of *Ubr4*-knockdown female *Drosophila*. As expected, similar hatchability rates were observed in both gender groups. The egg-hatching rate was significantly different between *Ubr4* knockdown and WT. The reasons might be the reduced fertility of the parents and the restricted development of the offspring. Firstly, knocking down the *Ubr4* males mated with normal females may lead to a decrease in the quality and quantity of eggs produced, which may be manifested by a decrease in egg survival and an increase in the rate of pseudo-eggs. Secondly, after *Ubr4* was knocked down, the genotypes of both offspring in the two gender groups were divided into four types, namely, (1) *tub-Gal4/+>UAS-Ubr4-RNAi/+*; (2) *tub-Gal4/+>+/+*; (3) *+/+>UAS-Ubr4-RNAi/+*; and (4) *+/+>+/+*, whereas the genotype of control groups was total *+/+>UAS-Ubr4-RNAi/+.* The egg-to-pupa hatchability can reflect the capacity of eggs to grow into pupae. Without *Ubr4* gene knockdown in the offspring of the control group, it can be explained why there was a significant difference in its egg hatchability. Indeed, differences may be observed between the *Ubr4* knockdown and the WT group if the quality of eggs cannot be ensured during production. Results showed that *Ubr4* knockdown resulted in greater inhibition of egg hatchability compared with the hatching rate of pupa-to-adult. The reasons behind this may be that the previous stages eliminated the quality of some of the poor eggs from developing into pupae. The remaining pupae, however, have sufficient capacity to hatch into adults. As previously mentioned, the offspring gene remained consistent irrespective of the parent genotype accounting for the lack of difference between both groups. It has been reported that *UBR4* encodes an E3 ubiquitination ligase (E3 enzyme), which regulates protein degradation and removes damaged or misfolded proteins from the cellular environment, thereby regulating cellular homeostasis (26). Protein error correction plays an important role in the development, reproductive inheritance, immunity, and endocrine and other important physiological processes of *Drosophila* (27–29).

It is now understood that *UBR4*, as an E3 enzyme, regulates the degradation of damaged or misfolded proteins (26). It is highly conceivable that *UBR4* gene knockdown disrupts the mechanisms that degrade erroneous proteins. We examined the protein expression in the *UBR4*-knockdown group and the WT control group to explore the mechanism behind the phenomenon. Among the GO terms that were most affected after knocking down *UBR4*, *Delta DNA polymerase complex*, *DNA polymerase complex*, and *Lipase activity* may be associated with the mechanism of male sterility after *UBR4* was knockdown. *DNA polymerase complex* has been reported to contribute to various disorders, including cancer and/or developmental defects (30). *Lipase activity* was associated with disorders of lipid metabolism, which has been reported to reduce semen quality and disrupted the blood–testis barrier integrity (31, 32). Downregulated expression of Pelo protein and upregulated Sfp65A protein was observed in the testis of *UBR4*-knockdown *Drosophila*. GO annotation indicated that Sfp65A and Pelo were associated with the reproductive process whereas CG11598 was related to biometabolic processes. Since the protein regulation ability of E3 ubiquitin ligase decreased following *UBR4* knockdown in *Drosophila*, Sfp65A protein exhibited a significant increase.

The increased proteins were likely damaged or misfolded and escaped from the regulatory process. *LIPA* encodes lysosomal acid lipase (LAL), which catalyzes the breakdown of LDL to produce free fatty acids and cholesterol (33). The study by JF-SHI showed that disruption of cholesterol homeostasis in semen affects semen quality and disrupts the blood–testis barrier, leading to reduced male fertility (31, 34).

Sfp is a non-sperm component of the ejaculate, which can increase male fitness in various aspects, encompassing sperm storage promotion, temporarily improving the female egg production rate, and decreasing female sexual receptivity (35) to ensure the quality and quantity of progeny production and reduced sperm competition (36–38). Therefore, the increase in damaged or erroneous Sfp65A proteins is associated with decreased ability to protect the normal reproductive ability of male *Drosophila*.

The Pelo protein has been documented in the mRNA surveillance pathway. Li et al. showed that Pelo forms complexes with Hbs1 to regulate multiple processes during spermatogenesis with the help of an mRNA surveillance pathway (39). Yang et al. indicated that Pelo played a significant role in silencing transposable elements (TEs) at the translation level, which was critical for genome integrity and primarily depended on Piwi proteins and associated RNAs (40). Both researchers concluded that the Pelo protein was important in stabilizing the quality of sperm and the structure of the genitals. On the other hand, the Sfp65A protein, which was significantly upregulated by inhibiting *UBR4* in *Drosophila*, has been associated with sperm development mediated by the Spliceosome pathway (41).

In the human cryptorchidism dataset (20), *UBR4*, *PELO*, and *LIPA* corresponded to the up- and downregulation of homologous genes in *Drosophila* in this experiment. This confirms that the effects of *UBR4* on male reproduction are likely to be reflected in human cryptorchidism. In terms of diagnostic efficacy, the AUC values for all three key genes were in the range of 0.810–0.880, indicating that these genes have moderate accuracy in diagnostic tests (42) and may be promising targets for the diagnosis of cryptorchidism.

To analyze the downstream genes predicting key genes, we screened six results (*MAPK14*, *NFKB1*, *MAPK13*, *MAPK11*, *MAPK9*, and *PIK3CD*) by single-gene GSEA and showed genes to be members of the IκB family, the JNK subfamily, and the MAPK family, a group of proteins that bind directly to the transcription factor NF-κB and regulate NF-κB activity, a key regulator of NF-κB activation (43, 44), and NF-κB has previously been shown to be associated with male sterility disorders (45). In GO and KEGG analyses, transitional secretion of interleukin-12 may cause abortion in women and abnormal sperm function in men (46, 47). The stress-activated protein kinase signaling cascade in BP annotation, and the stress-activated MAPK cascade and the MAP kinase activity in MF annotation, were all relative to the MAPK signaling pathway. By linking the results with the GSEA, the results suggest that activation of the MAPK signaling pathway has an important role in key genes such as *UBR4*, affecting male sterility. According to the current study, the MAPK-JNK signaling pathway regulates a variety of important physiopathological effects such as cell proliferation, differentiation, stress, and inflammatory responses (48, 49). Importantly, MAPK signaling regulates male fertility and plays multiple roles in various biological processes in germ cells and is closely linked to processes such as germ cell apoptosis and epididymal maturation (50). More specifically, the MAP4K4-JNK signaling pathway stimulates the proliferation of human spermatogonial stem cells and inhibits apoptosis, which is closely associated with male infertility (51). Thus, the MAPK pathway consisting of these downstream genes may be a key part of the mechanism linking the action of genes such as UBR4. In future studies, we will further explore the specific mechanism of action of UBR4 in the MAPK signaling pathway.

In conclusion, we provide preliminary evidence that after *UBR4* knockdown, male *Drosophila* fertility declines due to abnormal testis structure and low egg hatchability. Nonetheless, further studies are warranted to explore the underlying mechanisms.

## Data availability statement

The original contributions presented in the study are included in the article/supplementary material. Further inquiries can be directed to the corresponding authors.

## Author contributions

S-MX, J-XL, X-CC, J-DQ, and Y-LM designed the study and wrote the manuscript. C-QL, X-XZ and Y-ML performed the larva development, fertility and hatchability experiments. S-MX and J-XL performed testis dissection and analyzed the overall data. Q-WL and D-YH performed Bioinformatics analyses Q-WL and D-YH performed Bioinformatics analyses Q-WL and D-YH performed Q-WL and D-YH performed Bioinformatics analyses. All authors contributed to the article and approved the submitted version.
